# Neurophobia: why, how much, consequences and solutions

**DOI:** 10.15694/mep.2020.000003.1

**Published:** 2020-01-07

**Authors:** Virgilio Hernando-Requejo

**Affiliations:** 1 School of Medicine

**Keywords:** Neurophobia, neuroanatomy teaching, neurological examination learning, basic neurosciences, clinical neurosciences

## Abstract

This article was migrated. The article was marked as recommended.

The term *neurophobia* was defined by Jozefowicz as “a fear of the neural sciences and clinical neurology that is due to the students’ inability to apply their knowledge of basic sciences to clinical situations, leading to a paralysis of thought or action”. In this paper we review what we see as the key aspects of neurophobia. What gives rise to it? Notable among multiple causes are how basic and clinical neurosciences are taught, the peculiarities of neurological patient history, examination and differential diagnosis in the field, and how neurology and neurologists are seen from outside the field. We will also review the extent of the issue, for in view of its prevalence, many students will reject a specialty in increasing demand (as the incidence of neurological disorders will not cease to grow), along with its consequences: more patient referrals to neurology (owing to neurophobia or defensive medicine), or over-prescription of ancillary tests for diagnosis. Finally we will look at the solutions proposed, especially those aiming to bring about changes in the form and content of teaching, how the teaching of neurological examination and of new technologies is to be approached, and the use of those technologies as teaching aids.

## Definition

The term *neurophobia* was first used by Poser in 1959 ( Poser, 1959), though we owe its durable definition to Jozefowicz: “a fear of the neural sciences and clinical neurology that is due to the students’ inability to apply their knowledge of basic sciences to clinical situations, leading to a paralysis of thought or action” ( Jozefowicz, 1994). Neuroscience truly does engender anxiety in medical students, and this continues when on starting their clinical activity they are faced with neurological patients; if doctors are not trained in neurology, neurological examination and its interpretation will remain a real mystery.

## Causes

In this section we will consider what give rise to neurophobia. Notable among its multiple causes are how basic and clinical neurosciences are taught, the peculiarities of neurological patient history, examination and differential diagnosis in the specialty, and how neurology and neurologists are perceived from outside the field.

### Basic neuroscience

A common denominator in studies is to regard the separation of clinical and basic disciplines as the chief cause of neurophobia, especially if the latter are separated into neuroanatomy, neurophysiology, neuropathology and neuropharmacology ( Fantaneanu *et al.*, 2014; Pakpoor *et al*., 2014), for these subjects are normally taught by different specialists not linked to each other or to clinical practice. Moreover a lack of knowledge of basic neuroscience is one of the main reasons why medical students would reject a neurology residency ( Gupta *et al*., 2013). In his 1994 paper, Jozefowicz asserts that for students, separating the (basic) “science” and the (clinical) “art” makes the former irrelevant and the latter mystical ( Jozefowicz, 1994).

A 2018 study by Tarolli *et al*. with a sample of 3862 students and graduates, after a review of seven papers investigating the prevalence and drivers of neurophobia through surveys, identifies neuroanatomy as the main driver, due to its great difficulty. It is generally not taught by neurologists, so it is abstracted from clinical practice. And without this link, students do not know which parts of a detailed study of neuroanatomy are of clinical interest ( Tarolli & Jozefowicz, 2018). Indeed, many details of basic neuroscience are often irrelevant to clinical neurology ( Haines *et al.*, 2002).

It would be recommendable to consider developing a new approach in which the teaching of neurology to students begins with clinical signs and phenomenology, with explanations and reasons, rather than with basic disciplines lacking clinical focus ( Menken, 2002; Gupta *et al.*, 2013). If we could achieve a suitable vertical integration turning all these neuroscience disciplines into a single one, the problem would be mitigated ( Shelley *et al.*, 2018).

But the issue may be approached more aggressively, as by Zinchuk *et al*: Is neuroanatomy really important? If we assume that a “standard” resident has more knowledge of neuroanatomy in a medical degree than in a residency, it may be that it is not so important. They also use a driving analogy, i.e. that most of us drive without knowing how a car engine works ( Zinchuk *et al*., 2010).

It may be that part of the “blame” for the difficulty of basic neuroscience lies with the students tackling it. Knowledge requiring spatial orientation has to be acquired, and such skills are needed not so much for a career in medicine as for entering art school ( McCarron, 2014).

The “art” of reaching one of many possible diagnoses and the complexity of neurological examination

The weighting of clinical history in neurology is greater than in other fields (except for psychiatry). In numbers, according to Roshan & Rao (2000), in the making of diagnoses, the relative contributions are as follows:

• Clinical history, 78.6%
• Neurological examination, 8.2%
• Laboratory work and other ancillary tests, 13.2%

Despite technological advances, the key to neurological diagnosis is still the anatamo-clinical method. Diagnostic competence involves clinical reasoning, and eliciting a good clinical record takes some art and some science - as is also required for making initial hypotheses, dismissing some as new information is taken in, establishing a syndromic diagnosis and confirming it with examination, both through examination findings and after ruling out what is not expected to be found. In each case it is necessary to identify where the lesion is (neurolocalisation), its nature (neuropathology) and why it is there (aetiology), all of which is highly complex for students. It should be kept in mind that clinical experience and competence in neurology are analogous to the observation, investigation and inductive and abductive reasoning skills used to solve a crime mystery, as described in the fictional Sherlock Holmes stories ( Shelley *et al.,* 2018). We would considerably reduce neurophobia if we could make students realise that all one needs in order to get a grasp of neurology is to think logically ( Lim & Seet, 2008). Teaching clinical reasoning in medical degrees, rather than expecting it to be learned only with experience, would be highly useful.

Physical examination in neurology also requires special training; without the necessary skills this will be feared and avoided by non-neurologists. In the “TOS UK” study ( Nicholl *et al.*, 2012), when inpatients were questioned, 33% said they had not been examined with a tendon hammer, and 48% said they were not checked with an ophthalmoscope.

### Little course time in medical degrees, and little exposure to patients and neurological techniques

There is a distinct imbalance between the percentage of time devoted to neurology in medical degrees and the percentage in the burden of care generated by this specialty. Recently graduated doctors do not normally know how to handle a common, straightforward neurological case as would be recommendable ( Charles *et al.*, 1999; Bermejo-Pareja & Hernández-Gallego, 2007, Tarolli & Jozefowicz, 2018), prompting them to refer such patients to neurology from the outset, only aggravating so-called “neurological illiteracy”.

In a study by Gupta *et al.* (2013), 70% of students thought the teaching of neuropharmacology was deficient, and 67% believed their exposure to neurological patients was insufficient.

Pakpoor *et al.* (2014) survey 2877 medical students (61.6% women and 38.4% men) at 25 of the 31 faculties in the UK, i.e. some 7% of medical students across the country. Well, completing an undergraduate neurology course did not change their minds: neurology was the still seen as the most difficult specialty; 35% of students complained that the time devoted to neuroscience in the degree course was too little, 27% had not met a neurologist who inspired them, 26% did not feel confident that they knew what neurologists do, and 20% reported having not had the opportunity to carry out a clinical placement in neurology ( Pakpoor *et al.*, 2014).

Survey respondents much appreciate bedside tutorials with patients in order to learn how to elicit patient history and to make neurological examinations, and in particular they request training in outpatient care ( McCarron *et al*., 2014). Patients with rare syndromes are most likely to be admitted to neurology units, simply because they are hard to diagnose as outpatients. Medical students are exposed to such patients, especially complex ones, instead of getting to know “simpler”, more widespread and preventable disorders as they would in outpatient care ( McCarron *et al.* 2014). It seems that, contrary to what was previously thought, teaching the most “basic and mundane” parts of neurology promotes more interest than teaching its unusual and exclusive aspects ( McCarron *et al.*, 2014).

In the study by Gupta *et al.* (2013), the lack of exposure to and learning of neurology-specific diagnostic and therapeutic techniques was a major reason for not considering neurology as a future specialty, according to 57% of survey respondents. Most graduates know techniques such as electrocardiography, echocardiography and endoscopy, but have only slight knowledge of electroencephalography and electromyography. Exposure to these techniques in medical degrees should be encouraged, along with botulinum toxin injections or spinal tap. Code stroke systems may also attract many students ( Menken, 2002).

### Low standard of teaching and lack of mentors. Academic specialty with complex terminology and “diagnose and goodbye”

One modifiable factor of neurophobia for Fantaneanu *et al.* is the complexity of neurological terminology ( Fantaneanu *et al.*, 2014). Past neurologists identified and named more categories of disorders than in most other specialties ( McCarron *et al.*, 2014).

Neurology is a subject with difficult theory which is taught, according to pupils, by educators with a low teaching standard ( Flanagan *et al.*, 2007; Shelley *et al.*, 2018) and in many cases the educators are non-neurologists ( Tarolli & Jozefowicz, 2018).

The results of a systematic review ( McColgan *et al.*, 2013), analysing 16 randomised controlled trials, 9 non-randomised cohort/follow-up studies and 33 case series of educational interventions in neurology, using the Centre of Evidence‐based Medicine criteria and Kirkpatrick educational outcomes, showed no signs of high-quality education in neurology.

Contact with prestigious neurology educators notably promotes interest in the specialty ( Gupta *et al.* 2013). Teaching programmes are found to lack the figure of “neuro-mentor”, in order to stoke students’ interest and introduce them actively to neurological care and research ( Pakpoor *et al.*, 2014).

McCarron *et al.* (2014) analyse neurophobia in general practitioners - those who refer most neurological patients to the specialty. As compared with cardiology, endocrinology, gastroenterology, geriatrics, pneumology and rheumatology, neurology is the specialty that least interests them and of which they have the least mastery (with statistically significant findings for both aspects). Moreover 69% rated hospital teaching of neurology (and 61% for postgraduate teaching) as poor or very poor.

This gives rise to an image, an unfavourable stereotype of neurology, which in addition to the widespread view of neurologists as worn-out specialists (largely due to the neurophobia of others, generating an excessive workload), and of neurology as a specialty for “diagnose and goodby” (despite the many therapeutic advances undergone in this field), neurophobia is assured ( Tarolli & Jozefowicz, 2018). Neurologists should strive actively to be seen as therapists and not just as diagnosticians ( Gupta *et al.*, 2013).

### The “ivory tower” and the stigma of neurology

Perhaps neurologists contribute to neurophobia, for this allows us to be seen as select and brilliant ( Schon *et al.*, 2002).

An editorial in the British Medical Journal describes the issue as follows: “the neurologist is one of the great archetypes: a brilliant, forgetful man with a bulging cranium..who..talks with ease about bits of the brain you’d forgotten existed, adores diagnosis and rare syndromes, and - most importantly - never bothers about treatment.” ( Schon *et al.*, 2002). It may be that neurologists like the notion that neurology is a discipline suited only to “young Einsteins”. Such stereotypes only isolate neurology, and we run the risk of its social status deteriorating further in future decades ( Szirmai, 2012).

## Extent of the problem (amount of neurophobia)

In his paper that gave us a definition of neurophobia, Jozefowicz treats the issue as a disorder liable to be catalogued among mental illnesses. He does not base this on objective data, but this is not far from what has been demonstrated subsequently. He sees an incidence of 1:2 with men and women equally affected, with onset during training, and in some cases a familial predilection (neurophobic physician-parents) ( Jozefowicz, 1994). Subsequent studies calculate broadly that 18-47% of medical students suffer from neurophobia, with no difference between the sexes ( Shiels *et al.*, 2017), though for Kam *et al*. (2013) it is significantly more prevalent in women. There is also a degree of potential for contagion, as doctors with neurophobia seeing neurological patients can pass the condition on to their pupils ( Shelley *et al*., 2018).

We have many studies characterising neurophobia around the world ( Schon *et al.*, 2002; Flanagan *et al.*, 2007; Youssef, 2009; Zinchuk *et al.*, 2010; Gupta *et al.*, 2013; Kam *et al.*, 2013; Matthias *et al.*, 2013; Fantaneanu *et al.*, 2014; Lukas *et al.*, 2014; Pakpoor *et al.*, 2014; Abulaban *et al.*, 2015; Ramos *et al.*, 2016; Ansakorpi *et al.*, 2017).

Given that neurophobia is so prevalent, occurs at all levels of training and in all continents and persists over time, maybe it should be seen not as a disorder but as a natural state in medical students and doctors ( Fuller, 2012).

Authors are divided when making a prognosis for neurophobia. The most widely held view is that with the study of neuroscience it is mitigated, but Shiels *et al.* (2017), after surveying 446 students prior to studying neuroscience and 206 students afterwards, find that neurophobia grows significantly (p=0.035). Though knowledge clearly increases (p<0.001), there is no change either in interest in the specialty (p=0.327) or in its perceived difficulty (p=0.057).

In all events, the issue goes beyond the personal distress of those suffering from it and concerns increasing numbers of patients. Neurophobia leads to a rejection of all that has to do with a specialty which should be growing proportionally to the demand generated. A range of data evidence a growing imbalance between supply and demand: according to the WHO, neurological disorders exceed 6.3% of morbidity and 12% of mortality worldwide ( WHO, 2006). These figures will only increase as the years go by ( Menken *et al.*, 2000). If we focus on neurodegenerative disorders, in 2005-2030 the number of patients worldwide with Parkinson’s disease is set to double ( Dorsey *et al.*, 2007), and the prevalence of Alzheimer’s disease is expected to rise threefold by 2050 ( Johns, 2013). Despite this, the low percentage of doctors specialising in neurology in the US (less than 3%) has remained stable in recent decades. More than 20 years have passed since the disorder was described, and these issues subsist ( Tarolli & Jozefowicz, 2018).

## Consequences of neurophobia

One in ten general-practitioner (GP) consultations have a significant neurological component (Royal College of Physicians, 2011), and patients are referred increasingly to neurology for various reasons: defensive medicine, lower numbers of GPs, and naturally neurophobia in GPs (for many clinics regard neurology as the most interesting specialty, but also as the most complex one) ( Morrish, 2008; Ridsdale, 2009). Neurologists will receive most patents, both in hospital and in outpatient care, and other clinicians will gain no experience, which may make them increasingly afraid of such patients - and they communicate this fear to neurological patients, who feel more secure when attended to by neurologists ( Buchanan *et al.*, 2008). This insecurity does not require patients to have a rare syndrome but rather affects the treatment of slight and common neurological disorders. Matthias *et al.* (2013) survey 150 non-neurologists and 148 students, who see themselves as less secure when attending to patients with headache, paresthesia and dizziness than with other ailments.

As we see, we need a growing body of neurologists to cater for increasingly prevalent neurological disorders, and other physicians to be able to start a neurological assessment before resorting to a specialist ( Tarolli & Jozefowicz, 2018).

Yet neither the data nor the prospects are encouraging: the numbers of new neurologists are lower than for other specialties both in the US (where just 2.6% of medical degrees are specialised in neurology) and in Europe. The deficit of neurologists in the US is set to grow from 11% in 2013 to 19% in 2025. Even more so that of child neurologists, which in 2000 was already 20%. According to 2018 data, in India there are fewer than 1200 neurologists, with a ratio of 1 neurologist to 1,250,000 inhabitants ( Shelley *et al.*, 2018). The US, by contrast, has 1 neurologist for every 26,000 inhabitants, and Canada has 1 for every 53,000.

Some 20% of new referrals to neurology in the UK are for headache, and some 20% for seizure or epilepsy ( Wiles & Lindsay, 1996). In the UK around 20% of adult medical admissions in a district hospital are for neurological disorders, including strokes ( Morrow & Patterson, 1987). Of all disabilities, 28% are accounted for by neurological and psychiatric disorders ( Menken, 2002).

As the number of patients that may be referred to neurology is limited, appropriate selection is needed. In the UK, 97% of headaches are never referred to neurologists, but even so, the workload of neurologists limits their teaching capacity, closing a vicious circle of neurophobia.

Another adverse effect of neurophobia is that of “too much medicine”. Increasingly potent ancillary tests developed for neurological diagnostics, along with a lack of neurologists and neurophobia on the part of other physicians, will result in abusive, ill-directed use of such tests if physicians are not suitably trained, especially in primary care ( Bradley, 2010).

As we see, improving neurological skills in clinics would bring economic, legal and patient-safety benefits, as well as greater patient and doctor satisfaction ( Nicholl & Appleton, 2015).

## Strategies for mitigating neurophobia

Here we will review the most notable recommendations for mitigating neurophobia. These are strategies involving how neurology is taught, how to approach neurological examination and new technologies, and how to cast off the stereotypes detrimental to this specialty.

### Change in the form and content of teaching

#### Changes in the form of teaching

In medical degrees, neurology was traditionally taught by combining master classes, practical sessions, problem-based learning and clinicopathological lectures. Subsequently new teaching strategies have been added:

Team-based learning: this consists of small discussion groups, teamwork and immediate feedback to the educator. An initial assessment is made of the knowledge of each student and of the group, a clinical scenario to be worked upon is generated, and the educator is reported to on the session’s perceived outcomes ( McCarron, 2012). This allows clinical neurology to be better integrated with basic neuroscience through the application of recently learned physiopathology to clinical cases. Students make direct links between the basic and clinical spheres, and the relevance of what is learned is reinforced ( Tarolli & Jozefowicz, 2018).

This way of working is more student centred, and may teach the subject better and also enhance interpersonal communication and social skills. The best outcomes are generally achieved with academically at-risk students, but all improve ( Nieder *et al.*, 2005). Students are not left with unanswered questions, and there is more communication with educators.

Case-based learning (Shiels *et al.*, 2017): a clinical case is presented, first with basic patient history, and questions are asked about it and clinical changes proposed, and students have to reason through the changes. An expert educator guides the discussion. The aim is to ensure that student learning is focussed on continuous processing of information to solve a problem. It forces students to modify their ways of thinking and to combine new and previous knowledge. Basic and clinical neuroscience are integrated.

As regards practical study, bedside learning is useful if each student has a role in the medical team (responsibilities should be distributed between students and residents), and knows the patients (which will involve follow-up); otherwise students lose interest and feel left out ( Thompson Stone *et al.*, 2017). As we mentioned, contact solely with inpatients, with especially severe or rare disorders, may encourage neurophobia. It would be recommendable for students to rotate to outpatient clinics and so to be exposed to the common neurological pathologies which they will have to deal with at the end of their medical courses ( Thompson Stone *et al.*, 2017).

Students should keep being exposed to neurological patients so as not to relapse into neurophobia; one year passed away from such patients is enough for this to occur. Even after completing their degree course they should take continuing training in neurology ( Menken *et al.*, 1994). In view of the percentage of patients that will be seen by non-neurologists, Gupta *et al.* (2013) recommend the introduction of a short postgraduate course. In their study, 84% of students regard a 12-month neurology certification course as useful.

#### Changes in the content of teaching

The neurology syllabus should aim not to demonstrate that information has been stocked up but rather to show its contribution to health needs and social values. This would require different training in the various universities, based on the population’s characteristics, its culture (how do people accept neurological and psychiatric diagnoses?), the health system and its economic resources ( McCarron, 2012).

Standard teaching lacks the figure of a “one-on-one mentor” to promote the study of and liking for neurology. There should be programmes to promote this figure in undergraduate studies, though it already exists in some countries ( Shelley *et al.*, 2018).

When syllabuses are drawn up, as envisaged in the initial description of neurophobia, it is crucial to mix basic disciplines with clinical practice ( Youssef, 2009). Or, in other words, viewing basic aspects with a clinical perspective and clinical aspects with a sound basis. And clinical skills should be introduced in medical degrees as early as possible, so that students have time to practice them.

Another important task is to make a good selection of the basic neuroscience to be presented, always seeking a clinical application. Case-based teaching is a good way of combining, for example, neuroanatomy and clinical neurology (early clinical exposure) ( Shelley *et al.*, 2018).

Small-group teaching based on clinical cases in the preclinical years is useful, with guidance by residents and fellows (who thereby have to review basic neuroscience). Using selected patients in whole-class presentations has also proven to be effective ( Jozefowicz, 1994).

In the clinical years, basic-science educators should participate in conferences, so that their “anatomical thinking” may help students localise “clinical lesions” ( Jozefowicz, 1994).

When the clinical and basic spheres are joined, the latter becomes interesting (and pupils see that basic research affects clinical practice), and the former is better understood with basic knowledge ( Jozefowicz, 1994).

We find an example of the extent to which neurophobia may be mitigated through honing teaching strategy in Tarolli & Józefowicz (2018). At Rochester University there are 10 preclinical weeks, with courses in neuroanatomy, neurophysiology, neuropharmacology, neuropathology, psychopathology and psychopharmacology. The educators are enthusiastic and the residents get involved, and they review their results continuously. In the clinical phase there are 4 weeks of neurology and 4 of psychiatry. Thus 8.6% of their students in the past 5 years have studied neurology, as against 2.5% across the US (p<0.001). 89% of the physicians surveyed regarded the neurological training as excellent.

Finally, as regards the form and content of teaching, original methods have been proposed for improving clinical reasoning skills. Here are a few ( Shelley *et al.*, 2018):

• “Neuronovels”, such as books by the British neurologist Oliver Sacks.
• Non-medical fiction, such as “The Adventures of Sherlock Holmes”.
• Neurocinema: films linked to neurology such as “The Diving Bell and the Butterfly” (Locked in Syndrome), “Awakenings” (Encephalitis lethargica), and “Born on the Fourth of July” (spinal cord injury).

The aforesaid changes in teaching methods certainly require extra effort by professionals, who today, and if things do not change, often see more interest in spending time on research (for their CVs) or in providing care (for their income) ( Tarolli & Józefowicz, 2018).

Neurology organisations and societies should recognise the problem of neurophobia and deal with it, for example by standardising preclinical and clinical rotations, teaching times, etc. ( Tarolli & Józefowicz, 2018).

### Neurological examination

Neurological examination has great potential for intimidating students, who find, moreover, that there is no agreement between their educators on how it is to be taught. Most educators agree on conveying the importance of eliciting clinical history, but not on the role to be played by neurological examination in the diagnostic process. Two tendencies may be identified: systematic screening examination for all patients, or examination restricted and focussed case by case (to test a diagnostic hypothesis, and rule out others as appropriate). Students are confused if they see some neurologists proceeding in one way and others in another ( Counihan & Anderson, 2011).

In a study by Kamel *et al.* (2011), comparing screening and hypothesis-driven examination of patients with possible spinal focal deficits, it was found that hypothesis-driven examination had more chances of identifying abnormal signs, and with shorter examination times, but screening examination reduced the chances of misinterpreting normal results. They conclude that hypothesis-driven examination is superior for an acute focal process. But the study has major limitations, notably including that the students did not formulate the hypotheses but rather had checklists to search in, and did not use reflex hammers. In undergraduate teaching, it would be most advisable to combine both forms of examination ( Counihan & Anderson, 2011; McArron, 2012).

Students examining patients and then receiving feedback from them (on how the examination was made and their conduct during it) seem to have the best outcomes. This activity requires trained and even remunerated patients and is not without ethical limitations, especially given the physical and mental repercussions that it may have on such patients ( Park *et al.*, 2011; McArron, 2012).

Below we list some recommendations published regarding *how* and *when* in the teaching of neurological examination ( Tarolli & Józefowicz, 2018):

• Examinations in standard environments in the presence of physicians.
• From the preclinical phases of academic education, in order to differentiate normal signs from pathological ones early on and to enhance what will be learned later in the clinical phase.
• In the clinical phase, learning and practice of examination should continue, with positive feedback from educators.

### New technologies

Below we look at two approaches with regard to new technologies:

Used as support in teaching:

Today there is no question that Google helps residents to diagnose ( Elkind, 2009). Also video tutorials and online resources, in the absence of patients, help in honing neurological examination technique ( McCarron, 2012). But for the moment, online sources supplement but cannot replace conventional teaching methods ( Al-Shorbajin *et al.*, 2015).

There are ever more technology-based learning options that may be combined with “face-to-face” options. We shall see to what extent technology also teaches semiotics ( Ekstrand *et al.*, 2018).

The subject in which technology has been used most widely is neuroanatomy: using technology (websites, videos, etc.) to overcome the hurdle that trainees encounter when converting 2D images into 3D real brain structures. This is a difficult aspect of neuroanatomy and with 3D models it can be done more easily ( Ekstrand *et al.*, 2018).

Students get to know and learn to use new technologies in the sphere of medicine:

Medical technology is developing fast, and should be included in medical faculty education early on. More conceptual and integral teaching is recommendable, rather than teaching each technology in isolation. The idea is that students should learn the technological basis of medicine rather than to incorporate neurology-specific technology ( Tarolli & Józefowicz, 2018).

### Casting off the stereotypes on neurology, and fostering the educator-trainee relationship

There is a general tendency, even before starting a medical degree, to believe that neurologists are less satisfied than other specialists, that they are paid less and help their patients less (McArron, 2012; Fantaneanu *et al.*, 2014). These impressions may be overturned ( Matthias *et al.*, 2013), especially now that we have new more effective treatments.

In order to increase socialisation we have already mentioned teaching strategies involving work in small groups and close patient supervision, but there are further recommendations to be made (McArron, 2012; Tarolli & Józefowicz, 2018):

• Students should be able to access research projects, meet “neurological inspirers” and receive education further to that programmed in their degree.
• Offering courses to students who are interested, thereby extending their relationship with neurologists in time and helping them to consolidate (or otherwise) their interest in the specialty.
• Involving residents in undergraduate teaching, as they normally have more time, and from a generational viewpoint they are closer to students, so students are more at ease with them. At least in clinical rotations, teaching examination methods, but they would also be useful in the preclinical phase, as well as leading problem-based and team learning sessions. Residents in turn get involved in teaching and also learn.

## A brief respite: neurophilia

After so much talk of neurophobia we deserve a brief respite. Fuller (2012) defines “neurophilia” as fascination by neurology. It is widespread within both medicine and the general population, and may be a precondition to become a neurologist.

When choosing neurology as a specialty, what is that doctors most value? ( Gupta *et al.*, 2013):

• The specialty’s intellectual aspect
• The diagnostic challenges that it generates
• The type of patients involved
• Better quality of life (a controllable lifestyle), and time to spend with one’s family
• Inexpensive private practice

These are appealing aspects of the specialty. Among non-neurologists there are also indicators of neurophilia: in medical forums proportionally more neurological cases are presented than of other specialties, though the burden of care represented by such patients is lower. Moreover, books about neurological disorders represent 20% of those most widely sold in internal medicine, four times more than for any other medical specialty. Publishers and book-buyers seem to be prone to neurophilia. And the general public is also neurophile, judging by the large proportion of cases of this specialty appearing in the popular TV series “House” ( Fuller, 2012).

In his article Fuller seeks to reconcile the existence of so much neurophobia and so much neurophilia by arguing that it is an attractive specialty if the anxiety involved in diagnosis is removed. Combating neurophobia, if this is so, could reveal many neurophiles.

## Conclusion

With the available bibliography we may regard neurophobia as a global issue, a tendency with many medical students worldwide, giving rise to healthcare issues (owing to a lack of neurologists) of variable severity according to the country considered. The most significant causes of neurophobia have already been described, and valid solutions appear also to be known, yet the outlook is not encouraging. If we do not curtail neurophobia, the problem will only get worse: very few doctors being trained in neurology in a world in which neurological disorder will be increasingly prevalent.

Why, if the study of neurophobia began in 1994 and valid solutions have been proposed, has the situation not changed? The difficulty seems to lie in the complexity of the solutions put forward: a sweeping change is required in the form and content of neurological teaching, and, what may be even harder, active involvement by health institutions at various levels is required to give educators time, to ensure that they are suitably remunerated for teaching and to make this an activity that “looks good on a CV”. If in the future we manage to apply these complex solutions we may win a big victory over neurophobia. As Bertrand Arthur William Russell said, “the experience of overcoming fear is extraordinarily delightful”.

## Take Home Messages

• Neurophobia is a prevalent condition that leads to more patient referrals to neurology from general practitioners, and over-prescription of ancillary tests for diagnosis.
• The main causes of neurophobia are the uncoordinated teaching of basic and clinical neurosciences and the peculiarities of neurological examination and patient history.
• To minimize the prevalence of neurophobia, some changes in the form and content of teaching have been proposed, regarding theory, neurological examination and the use of new technologies as teaching aids.

## Notes On Contributors

V. Hernando-Requejo, Associate Professor, School of Medicine, CEU San Pablo University, Madrid, Spain, and Neurology Departments, University Hospitals Madrid Norte-Sanchinarro (Madrid) and Severo Ochoa (Leganés, Madrid, Spain). ORCID ID: https://orcid.org/0000-0003-1918-5335
