# Supplementary material for: Neurophobia: why, how much, consequences and solutions
Source: MedEdPublish (2016). 2020 Jan 7;9:3. [Version 1] doi: 10.15694/mep.2020.000003.1 (PMC10697450; doi:10.15694/mep.2020.000003.1)
Supplement: Peer review history

# Reviewer report: Reviewer response for version 1

**DOI:** 10.21956/mep.19969.r30356

**Published:** 2020-04-15

**Authors:** Ken Masters

**Affiliations:** 1 Sultan Qaboos University

This review has been migrated. The reviewer awarded 4 stars out of 5 An interesting paper on Neurophobia among medical students, addressing issues of why it occurs, how wide-spread it is, its consequences and possible solutions to the problem.Overall, this is a very well-written paper, and the author has nicely drawn on the literature to give an overview of the issues. I have one suggested improvement. The information is densely-packed, and that is fine (that can happen in a detailed narrative review). The problem is that each section is quite large, and the reader could get lost in some of the discussion. As a result, I would recommend that, at the end of each main section (“Causes”, “Extent of the problem (amount of neurophobia)” etc.), the author provide a brief synopsis of the section in order to ensure that the reader fully grasps what was said, before moving on to the next section.

Reviewer Expertise:

NA

No decision status is available

# Reviewer report: Reviewer response for version 1

**DOI:** 10.21956/mep.19969.r30354

**Published:** 2020-01-16

**Authors:** Barbara Jennings

**Affiliations:** 1 Norwich Medical School

This review has been migrated. The reviewer awarded 4 stars out of 5 In this narrative review, the author (Virgilio Hernando-Requejo) introduces us to the concept of ‘neurophobia’ and suggests a number of causes and problematic consequences of the phenomenon.The perceived fear of neurology and its underpinning basic sciences leads to a number of challenges that have been identified in the cited primary studies evaluating student experience, career plans, and neurology referrals. These include a reluctance for students and graduates to study and pursue a career in neurology; and limited mainstream diagnosis and management of neurological conditions in primary and non-specialist secondary care before specialist referral. These are big concerns for patient care, given the rising caseload of degenerative neurological conditions in an ageing population.The possible causes of ‘neurophobia’ are explored in detail, and Hernando-Requejo provides a thorough list of citations to back up the hypotheses or arguments. The range of issues identified in the list of problems includes familiar concerns for the educationalist: teaching anatomy and science in isolation/ without a clinical context; a lack of high quality educational programmes; a focus on rare conditions at the expense of the common and important; teaching being under-valued in comparison with other activities for the clinical academic. Other problems considered may be specific to neurology; e.g. the particular challenges associated with the rigor of the clinical reasoning required, and the spatial orientation skills required for neuroanatomy.The “strategies for mitigating neurophobia” read like a manifesto for the integrated, modern medical curriculum. With a focus on case based learning; early exposure to clinical skills; and by avoiding unnecessary cognitive overload, outcomes can be improved - and a more inspiring student- centred experience can emerge.I think the review could have been improved in two particular ways. First, when I read the abstract and definition I wondered whether this the author was describing a current problem, because of the reference to literature that was at least twenty five years old. I became much more convinced once I reached the section on page 5 (Consequences of Neurophobia) and the evidence for Neurology recruitment and service problems was presented. Perhaps this should have been introduced earlier in the review. Secondly, I wondered if the universal/generalisable nature of some of the challenges should have been articulated. After all, few specialists think that graduates are fully literate in, or prepared for their particular field of postgraduate medicine, be that genetics, haematology or indeed neurology.This review is very well-written and interesting; it reminded me of the value of thorough narrative (in comparison to systematic) reviews for providing a thought provoking and engaging overview of an important topic. The literature cited will be extremely useful for any medical educationalist working on the neurology undergraduate curriculum, or for postgraduate supervisors in this speciality. It is also a useful summary of important themes that will be of general interest to any faculty member working in clinical sciences or workforce planning initiatives.

Reviewer Expertise:

NA

No decision status is available

# Reviewer report: Reviewer response for version 1

**DOI:** 10.21956/mep.19969.r30355

**Published:** 2020-01-08

**Authors:** Felix Silwimba

**Affiliations:** 1 University of Lusaka

This review has been migrated. The reviewer awarded 5 stars out of 5 this is a very interesting article about neurophobia. I should add that the challenge is also about breaking the news to the patient and the family. because many a time the findings reveal a form of disability. some trainee doctors and qualified doctors are not ready to discuss disability with patients or family they would rather play it safe

Reviewer Expertise:

NA

No decision status is available
